# Development and Application of Multidimensional HPLC Mapping Method for *O*-linked Oligosaccharides

**DOI:** 10.3390/biom1010048

**Published:** 2011-12-14

**Authors:** Hirokazu Yagi, Erina Ohno, Sachiko Kondo, Atsuhiro Yoshida, Koichi Kato

**Affiliations:** 1 Graduate School of Pharmaceutical Science, Nagoya City University, 3-1 Tanabe-dori, Mizuho-ku, Nagoya 467-8603, Japan; E-Mails: hyagi@phar.nagoya-cu.ac.jp (H.Y.); e98731515@yahoo.co.jp (E.O.); Kondou.Sachiko@glyence.co.jp (S.K.); 2 Graduate School of Medical Sciences and Medical School, Nagoya City University, Kawasumi-1, Mizuho-cho Mizuho-ku, Nagoya 467-8601, Japan; E-Mail: atsuhiro@med.nagoya-cu.ac.jp; 3 Institute for Molecular Science and Okazaki Institute for Integrative Bioscience, National Institutes of Natural Sciences, 5-1 Higashiyama Myodaiji, Okazaki 444-8787, Japan; 4 GLYENCE Co., Ltd., 2-22-8 Chikusa, Chikusa-ku, Nagoya 464-0858, Japan; 5 The Glycoscience Institute, Ochanomizu University, 2-1-1 Ohtsuka, Bunkyo-ku, Tokyo 112-8610, Japan

**Keywords:** *O*-glycans, HPLC map, glycosylation profiling, sulfated oligosaccharide, IgA

## Abstract

Glycosylation improves the solubility and stability of proteins, contributes to the structural integrity of protein functional sites, and mediates biomolecular recognition events involved in cell-cell communications and viral infections. The first step toward understanding the molecular mechanisms underlying these carbohydrate functionalities is a detailed characterization of glycan structures. Recently developed glycomic approaches have enabled comprehensive analyses of *N*-glycosylation profiles in a quantitative manner. However, there are only a few reports describing detailed *O*-glycosylation profiles primarily because of the lack of a widespread standard method to identify *O*-glycan structures. Here, we developed an HPLC mapping method for detailed identification of *O*-glycans including neutral, sialylated, and sulfated oligosaccharides. Furthermore, using this method, we were able to quantitatively identify isomeric products from an *in vitro* reaction catalyzed by *N*-acetylglucosamine-6*O*-sulfotransferases and obtain *O*-glycosylation profiles of serum IgA as a model glycoprotein.

## 1. Introduction

Glycosylation is one of most ubiquitous post-translational modifications. Carbohydrate moieties, which are typically found on asparagine or serine/threonine residues, are associated with an increase in solubility and stability of proteins, structural integrity of protein functional sites, and mediation of biomolecular recognition events involved in cell-cell communications and viral infections [1,2]. Since glycans affect the serum half-life of proteins and functional protein–protein interactions, glycosylation is currently considered to be a crucial factor in the design and development of biopharmaceuticals [3,4,5]. To address the detailed molecular basis of the functional roles of protein glycosylation, the first step is identifying the glycan structures expressed on the proteins [6,7,8,9,10]. Recently developed glycomic approaches using chromatographic and mass spectrometric (MS) techniques have enabled comprehensive analyses of *N*-glycosylation profiles [11,12]. For example, a multidimensional HPLC mapping method has been developed for quantitative *N*-glycosylation profiling at molecular, cellular, and tissue levels, enabling isomeric *N*-glycan structures [13,14]. In this method, identification of individual *N*-glycans is based on their elution positions on three types of HPLC columns. The accumulated HPLC data of approximately 500 different *N*-glycans are now available by using the web application GALAXY (http://www.glycoanalysis.info/galaxy2/ENG/index.jsp) [15], and the applicability of this method has been extended to sialylated, glucronylated, and sulfated oligosaccharides [16,17,18].

However, few reports describe the detailed *O*-glycosylation profiles with linkage information due to the lack of widespread standard methods for unambiguous identification of *O*-glycan structures [19,20,21]. The HPLC elution conditions employed in the current GALAXY protocols are not applicable to the profiling of *O*-glycans, because they frequently include smaller saccharides, e.g., mono- and di-saccharides, in contrast to the generally larger *N*-glycans. In view of this situation, we herein attempted to develop HPLC-based profiling of *O*-glycans for their detailed structural identification. By chromatographic and mass spectrometric analyses in conjunction with several exoglycosidase treatments *in vitro,* we successfully collected HPLC data for 27 different *O*-glycans including neutral, sialyl, and sulfated oligosaccharides, which were isolated from natural sources and/or by *in vitro* enzymatic reactions. By applying this extended HPLC map, we have obtained *O*-glycosylation profiles of serum immunoglobulin A (IgA) as a model glycoprotein. Furthermore, we characterized branch specificity in the sulfation reaction catalyzed by human *N*-acetylglucosamine-6*O*-sulfotransferases (GlcNAc6ST)-1.

## 2. Experimental

### 2.1. Materials

Materials used for the experiments were purchased from the sources indicated below: Glycoamidase A from sweet almond, β-galactosidase and β-*N*-acetylhexosaminidase from jack bean were purchased from Seikagaku Kogyo Co. (Tokyo, Japan). α-Galactosidase from green coffee bean was purchased from Oxford Glycosystems Inc. (Bedford, MA, USA) (currently available in Prozyme (Hayward, CA, USA)). α-Sialidase from *Arthrobacter ureafaciens* was purchased from Nacalai Tesque (Kyoto, Japan). α2,3-Sialidase from *Salmonella typhimurium* was purchased from Takara Bio Inc. (Otsu, Japan). Recombinat α2,3-sialyltransferase and α2,6-sialyltransferase were purchased from Calbiochem (San Diego, CA, USA). Colostrum IgA, porcine stomach mucin, trypsin, and chymotrypsin were purchased from Sigma Chemical Co. (St. Louis, MO, USA).

2-Aminopyridine-derivatized (PA) isomalto-oligosaccharides were prepared from glucose oligomers (1-20) (Seikagaku Kogyo Co., Tokyo, Japan), fucose (Fuc), galactose (Gal), *N*-acetylgalactosamine (GalNAc) (Seikagaku Kogyo Co., Tokyo, Japan), glucose (Glc), *N*-acetylglucosamine (GlcNAc), mannose (Man), Galβ1-3GalNAc (Calbiochem, Schwalbach, Germany), and Galβ1-3(Fucα1-2)GalNAc. Four types of *O*-glycosylated peptides—Galβ1-4GlcNAcβ1-6(Neu5Acα2-3Galβ1-3)GalNAcα 1-peptide (AHGVT*SAPDTRK; asterisks indicate glycosylation sites)-FAM (5-carboxyfluorescein), Galβ1-4GlcNAcβ1-6(Galβ1-3)GalNAcα1-peptide-FAM, GlcNAcβ1 -6(GlcNAcβ1-3Galβ 1-3)GalNAcα1-peptide-FAM, and Neu5Acα2-6(Galβ1-3)GalNAc-peptide-FAM were purchased from GlycoGene (Tsukuba, Japan).

### 2.2. Purification of IgA from Human Serum

Human serum (1 mL) was diluted in 10 mL of 0.01 M phosphate buffer (pH 7.4) containing 0.15 M NaCl and absorbed on jacalin-agarose columns (1 mL) (Thermo Scientific, San Jose, CA, USA). After the column was thoroughly washed with 10 mL of 0.01 M phosphate buffer (pH 7.4) containing 0.15 M NaCl and 0.8 M glucose, lectin-binding proteins were eluted with 10 mL of 0.01 M phosphate buffer (pH 7.4) containing 0.1 M melibiose as described previously [22]. After the eluate was concentrated using an AMICON Ultra-15 centrifugal filter unit (Millipore, Billerica, MA, USA), serum IgA was purified with a Superose 12 gel filtration column (GE Healthcare, Uppsala, Sweden) equilibrated with 0.01 M phosphate buffer (pH 7.4) containing 0.15 M NaCl. The purified IgA was desalted with a PD-10 column (GE Healthcare) according to the manufacturer's instructions and then lyophilized for glycosylation profiling.

### 2.3. Sulfation Reaction by GlcNAc6ST-1

COS7 cells grown in 75 cm^2^ culture flasks (Corning, Corning, NY) were transfected with 10 μg of relevant plasmid, pcDNA-GlcNAc6ST-1 [23] using Lipofectamine Plus (Invitrogen, Carlsbad, CA, USA) according to manufacturer's instructions. After 24 h of culture in Dulbecco's modified Eagle's medium (DMEM) containing 10% fetal calf serum, the medium was replaced with DMEM containing 2% IgG-free fetal calf serum. The cells were further cultured for 96 h. Subsequently, the culture medium was collected and concentrated to 1 mL using Amicon Ultra-15 (Millipore). The recombinant protein A-fused GlcNAc6ST-1 expressed in the medium was adsorbed to IgG-Sepharose (20 μL resin/1 mL of culture medium) at 4 °C for 3 h. The resin was collected by centrifugation and washed three times with 50 mM Tris-HCl (pH 7.5). Finally, the resin was suspended in 20 μL of 50 mM Tris-HCl (pH 7.5) and used for enzymatic reaction. The glycopeptide GlcNAcβ1-6(GlcNAcβ1-3Galβ1-3)GalNAcα1-peptide-FAM was utilized as an acceptor substrate. The standard reaction mixture was composed of 1 μmol of Tris-HCl (pH 7.5), 0.4 μmol of MnCl_2_, 0.08 μmol of AMP, 24 μmol of NaF, 50 pmol of glycopeptide, 300 pmol of 3′-phosphoadenosine 5′-phosphosulfate, 0.1% Triton X-100, and 20 μL of the fusion protein suspension in a final volume of 40 μL. After incubation at 37 °C for 1, 5, 24, and 48 h, the individual reaction mixtures were applied to a TSK gel ODS-80s HPLC column (TOSOH, Tokyo, Japan) at a flow rate of 1.0 mL/min at 25 °C using two solvents: G and H. G comprised water containing 0.05% trifluoroacetic acid and H comprised acetonitrile-2-propanol (2:1, v/v) containing 0.05% trifluoroacetic acid. The column was equilibrated with 90% solvent G and 10% solvent H. The time for gradient elution was 0–40 min with a linear gradient of 10%–15% D. The glycopeptides were detected by fluorescence using excitation and emission wavelengths of 492 and 520 nm, respectively.

### 2.4. Liberation of O-glycans from Glycoproteins

The *O*-glycans were released from glycoproteins and glycopeptides by β-elimination using hydrazine for a convenient modification with 2-aminopyridine. Lyophilized glycoproteins (∼250 μg) or glycopeptides (∼5 μg) were dissolved in 1 mL of distilled anhydrous hydrazine with a water content of less than 1% (v/v) in 10 mL glass tube, incubated at 60 °C for 6 h and quenched by 9 mL of 50 mM ammonium acetate buffer (pH 7) with slight modification of the previous literature [24]. The excess hydrazine, peptides, and other reagents were removed and *N*-acetylated using a graphite carbon column (GL-Pak Carbograph, GL Science, Tokyo, Japan) according to the literature [25]. The hydrazine solution was mixed with 3 mL of 50 mM ammonium acetate buffer (pH 7) and loaded onto the GL-Pak Carbograph column. After the column was washed with 15 mL of 50 mM ammonium acetate buffer (pH 7.0), the released glycans were eluted with 5 mL of a mixture of 50 mM ammonium acetate buffer (pH 7.0):acetonitrile containing 2% acetic anhydride (40:60).

### 2.5. Pyridylamination

The released *O*-linked saccharides, as well as those commercially obtained, were labeled with 2-aminopyridine as described previously [26]. Ten volumes of acetonitrile were added to one volume of reaction mixture. The excess PA reagents were removed with a MonoSpin NH2 desalting column (GL Science). After the column was equilibrated with 200 μL of acetonitrile, the PA reaction mixture was loaded onto the column. The column was washed with acetonitrile three times. Then, the PA-saccharides were eluted with 100 μL of water and subsequently dried under vacuum.

### 2.6. HPLC and MS Analyses

Three types of HPLC columns were used for the separation of PA-saccharides. In each step, PA-saccharides were detected by fluorescence using excitation and emission wavelengths of 310 and 380 nm, respectively. The PA-saccharide mixture was firstly separated on a Mono-Q HR 5/5 anion-exchange column (GE Healthcare) at 30 °C with a flow rate of 1.0 mL/min using two solvents, A and B. Solvent A was aqueous ammonia (pH 9.0) and solvent B was a 50 mM ammonium acetate solution (pH 9.0). The column was equilibrated with solvent A. The gradient elution parameters were 0–3 min, linear gradient 0%–12% B; 3–17 min, linear gradient 12%–40% B; 17–22 min, linear gradient 40%–100% B. Each oligosaccharide was separated according to its anionic charges.

In the second step, each fraction separated from the Mono-Q column was collected, evaporated, and then applied to a Decelosil C30-HG-5 (C30) column (Nomura Chemical Co., Ltd., Seto, Japan). Elution was performed at a flow rate of 1.0 mL/min at 25 °C using two solvents, C and D. Solvent C was 0.1 M ammonium acetate buffer (pH 6.0) containing 0.01% 1-butanol and solvent D was 0.1 M ammonium acetate buffer (pH 6.0) containing 1% 1-butanol. The column was equilibrated with solvent C. The gradient elution parameters were 0–51 min, linear gradient 0%–50% D and 51–63 min, linear gradient 50%–100% D.

In the third step, individual peak fractions from the C30 column were isolated using a TSK gel amide-80 size fractionation column (TOSOH). In this system, two solvents were used at 25 °C. Solvent E was composed of 3% acetic acid in water with triethylamine (pH 7.3) and acetonitrile, 15:85 by volume. Solvent F was composed of 3% acetic acid in water with triethylamine (pH 7.3). The column was equilibrated with solvent E. The gradient elution parameters were 0–5 min, linear gradient 0%–20% F and 5–17 min, linear gradient 20%–44% F.

The HPLC elution times were represented by glucose units (GUs) on the columns calibrated with a PA-derivatized isomalto-oligosaccharides mixture. The structures of the PA-saccharides were characterized by HPLC mapping in conjunction with exoglycosidase treatments and matrix-assisted laser desorption/ionization time of flight (MALDI-TOF-MS) analysis using a MALDI-TOF-MS spectrometer (AXIMA-CFR; Shimadzu, Kyoto, Japan). Collision-induced dissociation spectra of PA-oligosaccharides were acquired using a MALDI-quadrupole ion trap (QIT)-TOF-MS spectrometer (AXIMA-QIT; Shimadzu). All analytical procedures used in this work, including sulfation, sialylation, several glycosidase treatments, and MALDI-TOF-MS analyses have been described previously [16,17,27,28,29].

## 3. Results and Discussion

### 3.1. Collection of HPLC Data of O-linked Saccharides

**Figure 1:**
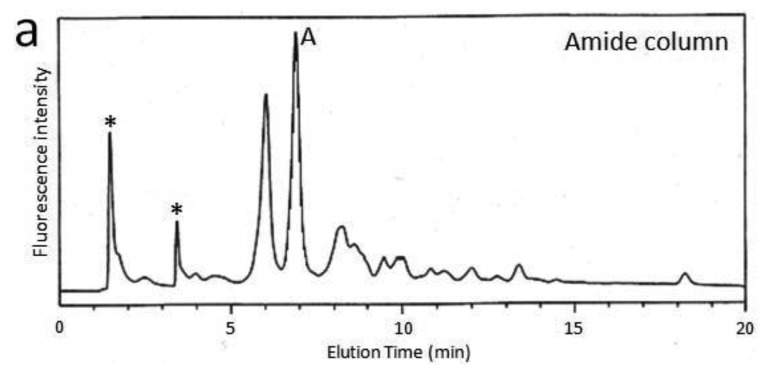

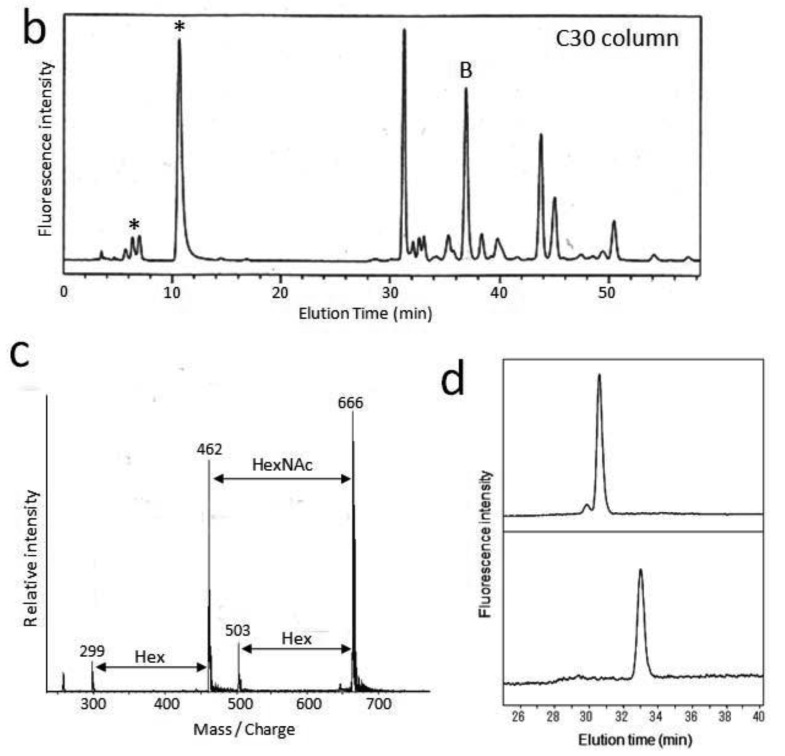
Isolation and identification of an *O*-glycan derived from porcine stomach mucin. (**a**)Chromatogram of PA-glycans derived from mucin on an amide column; (**b**) Chromatograms of the PA-glycans corresponding to fraction A on the C30 column; (**c**) MALDI-QIT-TOF-MS/MS spectra of the PA-glycan corresponding to fraction B. Precursor ion was *m/z* 666 as protonated ion; (**d**) Chromatograms of the PA-glycan corresponding to fraction B on the C30 column (upper) before and (lower) after β1,3-galactosidase treatment. The asterisk indicates the fractions containing no detectable PA-saccharide.

First, we attempted to make an HPLC map of the standard PA-saccharide. The PA tag was attached to the commercially obtained saccharides Fuc, Gal, GalNAc, Glc, GlcNac, Man, Galβ1-3GalNAc, and Galβ1-3(Fucα1-2)GalNAc. In addition, four types of *O*-glycosylated peptides were treated with hydrazine, and the released oligosaccharides were labeled with 2-aminopyridine. The PA-saccharides thus prepared were subjected to amide and C30 columns to record their elution times (Table 1). Furthermore, pyridylaminated *O*-glycans were prepared from colostrum IgA and porcine stomach mucin, and their structures were identified by chromatographic analyses combined with exoglycosidase treatments and MALDI-TOF-MS. The structural identification of these *O*-glycans would be exemplified by a glycan derived from mucin. Since no sialylated *O*-glycans were detected in the glycosylation profile of mucin on a Mono-Q column (data not shown), the PA-glycans were directly applied to an amide column. Figure 1a shows the *O*-glycosylation profile of the mucin on the amide column in which two major *O*-glycans were found. Then, fraction A was applied to a C30 column, giving rise to several peaks including B (Figure 1b). The elution times of the PA-*O*-glycan in fraction B are represented as 3.2 GU on the amide column and 4.6 GU on the C30 column. The molecular mass of this glycan was determined by MALDI-TOF-MS analysis as 665 Da, which corresponds to (Hex)_1_(HexNAc)_2_PA (Figure 1c). The fragment ions indicated that the PA-*O*-glycan exhibits the branching structure Hex-(HexNAc-)HexNAc-PA. Finally, the glycan corresponding to fraction B was treated with β1,3-galactosidase and then applied to a C30 column, giving rise to a new fraction. The elution time of the glycan corresponding to this fraction coincided with that of the reference PA-glycan, GlcNAcβ1-6GalNAc-PA. On the basis of all these data, we concluded that the glycan corresponding to fraction B was Galβ1-3(GlcNAcβ1-6)GalNAc-PA.

**Table 1:** HPLC and mass sprctrometric (MS) data of PA-*O*-glycans.

**PA-Saccharides**	**GU (amide)**	**GU (C30)**	**Molecular mass (Da) *a***
GalNAc-PA	0.8	3.0	300
Man-PA	0.8	0.8	259
GlcNAc-PA	1.0	2.7	300
Gal-PA	1.2	0.4	259
Fuc-PA	0.7	1.5	243
Galβ1-3GalNAc-PA	1.7	2.4	462
GlcNAcβ1-6GalNAc-PA	2.4	6.3	503
Galβ1-3(Fucα1-2)GalNAc-PA	2.4	8.5	608
GlcNAcβ1-3Galβ1-3(GlcNAcβ1-6)GalNAc-PA	3.0	2.9	868
Galβ1-3(Galβ1-4GlcNAcβ1-6)GalNAc-PA	3.4	4.0	827
Neu5Acα2-3Galβ1-3(Galβ1-4GlcNAcβ1-6)GalNAc-PA	3.2	4.3	1118
Galβ1-3(Neu5Acα2-6)GalNAc-PA	1.6	2.3	753

a Average mass calculated from the *m/z* values of [M+H]^+^, [M+Na]^+^, and [M-H]^–^ ions for PA-saccharides.

**Figure 2:**
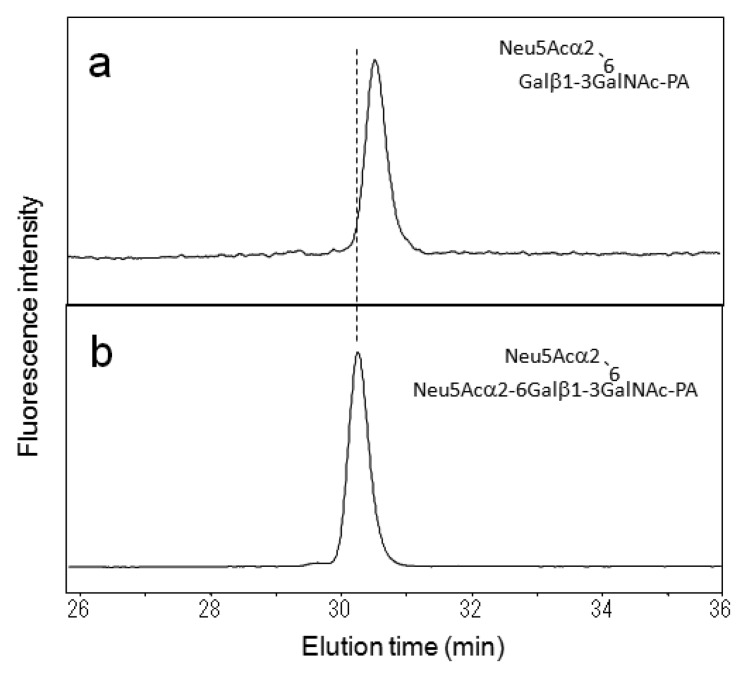
Identification of the disialyl PA-saccharide produced by the reaction catalyzed by α2,6-sialyltransferase. Chromatograms of (**a**) the precursor Galβ1-3(Neu5Acα2-6)GalNAc-PA; and (**b**) the reaction product Neu5Acα2-6Galβ1-3(Neu5Acα2-6)GalNAc-PA on the C30 column.

**Figure 3:**
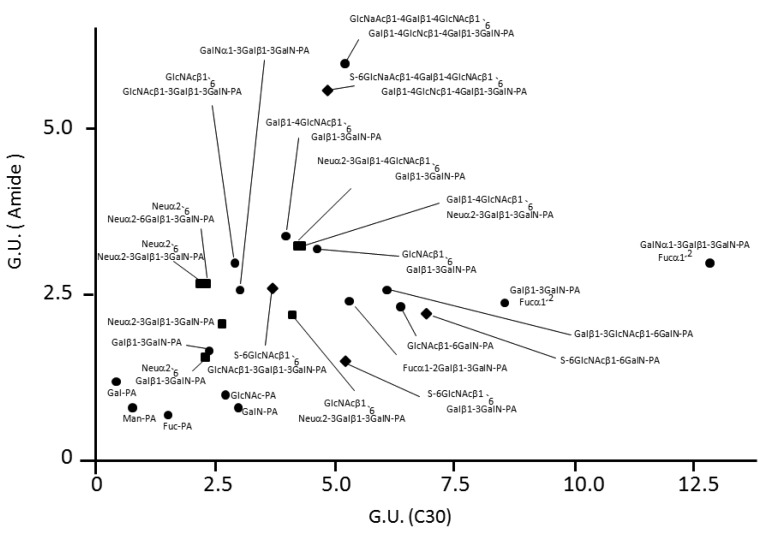
HPLC map of *O*-glycans. •, neutral; ▪, sialylated; ♦, sulfated glycans. Key: Gal, galactose; Glc, Glucose; GlcNAc, *N*-acetylglucosamine; GalN, *N*-acetylgalactosamine; Man, mannose; Fuc, fucose; S, sulfate; Neu, *N*-acetylneuraminic acid.

With similar methodology, we identified eight types of *O*-glycans derived from mucin and IgA glycoproteins and recorded their elution times (Table 2). Using the HPLC data as a guide, these *O*-glycans could be strategically collected from glycoproteins and further derivatized by glycosidase glycosyltransferase and sulfotransferase treatments *in vitro,* giving rise to a variety of standard PA-oligosaccharides. For example, the mono-sialyl PA-oligosaccharide Galβ1-3(Neu5Acα2-6)GalNAc-PA was treated with α2,6-silayltransferase, giving rise to di-sialyl PA-oligosaccharide Neu5Acα2-6Galβ1-3(Neu5Acα2-6)GalNAc-PA, which was eluted differently from the reaction precursor on the C30 column (Figure 2). Similarly, we collected the HPLC data of seven kinds of PA-*O*-glycans (Table 3). Finally, we made an HPLC map containing 16 neutral, seven sialylated, and four sulfated *O*-glycans (Figure 3).

**Table 2:** HPLC and MS data of PA-*O*-glycans derived from colostrum IgA and porcine stomach mucin.

**PA-Saccharides**	**GU (amide)**	**GU (C30)**	**Molecular mass (Da) *a***	**Source**
Fucα1-2Galβ1-3GalNAc-PA	2.4	5.3	608	Porcine stomach mucin
Galβ1-4GlcNAcβ1-4Galβ1-3(GlcNAcβ1-4Galβ1-4GlcNAcβ1-6)GalNAc-PA	6.0	5.2	1395	Porcine stomach mucin
GalNAcα1-3(Fucα1-2)Galβ1-3GalNAc-PA	3.0	12.8	811	Porcine stomach mucin
Galβ1-3GlcNAcβ1-6GalNAc-PA	2.6	6.1	665	Porcine stomach mucin
GalNAcα1-3Galβ1-3GalNAc-PA	2.6	3.0	665	Porcine stomach mucin
Galβ1-3(GlcNAcβ1-6)GalNAc-PA	3.2	4.6	665	Porcine stomach mucin
Neu5Acα2-3Galβ1-3GalNAc-PA	2.1	2.6	753	Colostrum IgA
Neu5Acα2-3Galβ1-3(Neu5Acα2-6)GalNAc-PA	2.7	2.2	1044	Colostrum IgA

a Average mass calculated from the *m/z* values of [M+H]^+^, [M+Na]^+^, and [M-H]^–^ ions for PA-saccharides.

**Table 3:** HPLC and MS data of PA-*O*-glycans produced *in vitro* by derivatisation of the neutral *O*-glycans listed in Table 1 and Table 2.

**PA-Saccharides**	**GU (amide)**	**GU (C30)**	**Molecular mass (Da) *a***
Neu5Acα2-3Galβ1-3(GlcNAcβ1-6)GalNAc-PA	2.2	4.1	956
Galβ1-3(Neu5Acα2-3Galβ1-4GlcNAcβ1-6)GalNAc-PA	3.2	4.2	1118
Neu5Acα2-6Galβ1-3(Neu5Acα2-6)GalNAc-PA	2.7	2.3	1044
HSO_3_-6GlcNAcβ1-6GalNAc-PA	2.2	6.9	583
Galβ1-4GlcNAcβ1-4Galβ1-3(HSO_3_-6GlcNAcβ1-4Galβ1-4GlcNAcβ1-6)GalNAc-PA	5.6	4.9	1475
GlcNAcβ1-3Galβ1-3(HSO_3_-6GlcNAcβ1-6)GalNAc-PA	2.6	3.7	948
Galβ1-3(HSO_3_-6GlcNAcβ1-6)GalNAc-PA	1.5	5.2	745

a Average mass calculated from the *m/z* values of [M+H]^+^, [M+Na]^+^, and [M−H]^−^ ions for PA-saccharides.

The HPLC map thus established facilitates the quantitative *O*-glycosylation profiling with discriminating isomeric structures of *O*-glycans, which would be difficult to perform by MS-based approaches. The HPLC-based *O*-glycosylation profiling methods so far reported need much longer elution times (more than 2 h) or employ different elution conditions between neutral and acidic *O*-glycans [19,20]. Our developed HPLC map is able to deal with neutral and anionic *O*-glycans (including sulfated *O*-glycans whose HPLC data have not been reported previously) with the same protocol using a shorter elution time (within 1 h) and therefore would be advantageous in comparison to previously reported methods.

### 3.2. Branch Specificity of GlcNAc6ST-1

The HPLC map thus established will facilitate structural identification of sulfated *O*-glycans. To date, five types of sulfotransferases (termed GlcNAc6STs) have been reported to catalyze a sulfate group on the C6 position of GlcNAc residues [30]. Although spatio-temporal expression patterns of these enzymes have been extensively characterized, their reaction specificities are not fully understood. We herein applied the developed HPLC data to examination of the branch specificity of enzymatic sulfation catalyzed by human GlcNAc6ST-1, which was expressed by COS7 cells as a fusion protein with protein A [23].

**Figure 4:**
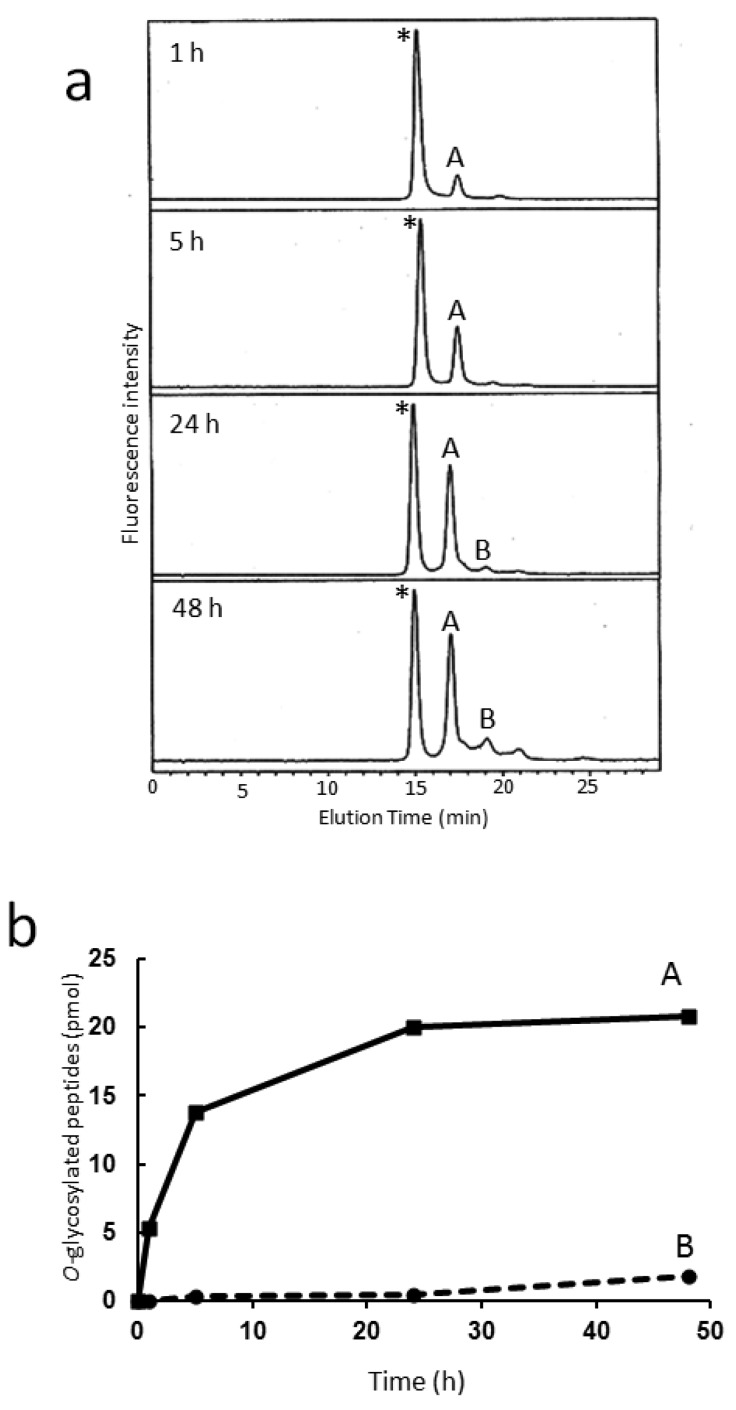
HPLC-based characterization of branch specificity of GlcNAc6ST-1. (**a**) Time-dependent change of the HPLC profiles on the ODS column for products resulting from glycopeptides possessing two terminal GlcNAc residues during the sulfation reaction catalyzed by the recombinant GlcNAc6ST-1. The asterisk indicates the fractions containing the substrate *O*-glycosylated peptide: GlcNAcβ1-6(GlcNAcβ1-3Galβ1-3)GalNAcα1-peptide-FAM; (**b**) Time course of the amounts of the resultant glycopeptides corresponding to fraction A (solid line) and B (dashed line). The *O*-glycan structures of fractions A and B were identified as GlcNAcβ1-6(HSO_3_-GlcNAcβ1-3Galβ1-3)GalNAc and HSO_3_-GlcNAcβ1-6(GlcNAcβ1-3Galβ1-3)GalNAc, respectively.

Figure 4a shows the time-dependent change of reverse-phase HPLC elution profiles for the reaction mixture of the *in vitro* sulfation catalyzed by this recombinant enzyme using a fluorescent glycopeptide, GlcNAcβ1-6(GlcNAcβ1-3Galβ1-3)GalNAcα1-peptide-FAM, as an acceptor. MALDI-TOF-MS indicated that two reaction products, A and B, were isomeric glycopeptides that possessed a single sulfate group. For unambiguous identification of the isomeric structures of sulfated *O*-glycans, fractions A and B were subjected to the developed HPLC mapping. As a result, the PA-glycans derived from fractions A and B were identified as GlcNAcβ1-6(HSO_3_-GlcNAcβ1-3Galβ1-3)GalNAc-PA and HSO_3_-GlcNAcβ1-6(GlcNAcβ1-3Galβ1-3)GalNAc-PA, respectively. This result clearly indicates that GlcNAc6ST-1 selectively catalyzed sulfation at the β1-6-linked GlcNAc residue in comparison with the remaining β1-3-linked GlcNAc residue, consistent with the previous report that preferential sulfation occurs with core 2 (GlcNAcβ1-6(Galβ1-3)GalNAc) rather than core 3 (GlcNAcβ1-3Galβ1-3GalNAc) as the acceptor [23]. Thus, our HPLC map is a useful tool for detailed characterization of substrate and reaction specificities of glycosyltransferases and sulfotransferases, leading to a better understanding of their detailed functional roles.

### 3.3. O-glycosylation Profiling of Serum IgA

**Figure 5:**
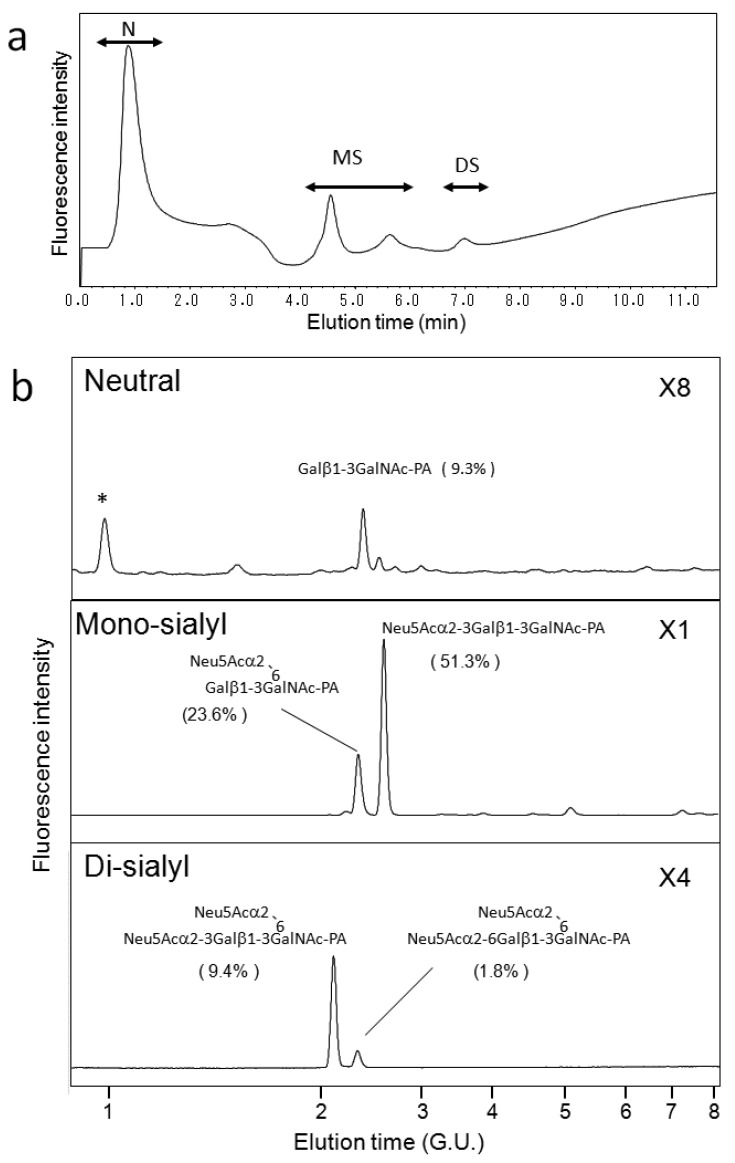
HPLC profiles of PA-*O*-glycans derived from human serum IgA. (**a**) Chromatogram of PA-*O*-glycans derived from serum IgA on a Mono-Q column. The PA-glycan mixture was separated according to sialic acid contents. N, neutral; MS, mono-sialyl; DS, di-sialyl; (**b**) Chromatograms of the neutral, mono-sialyl, and di-sialyl fractions on a C30 column. The structures of PA-*O*-glycans in each fraction were identified on the basis of the HPLC map. Molar percent of the *O*-glycan content in the IgA sample was calculated based on the peak areas. The structure and incidence of major *O*-glycans are shown on the profiles. Asterisks indicate the peaks derived from melobiose used for IgA purification.

We also applied our HPLC map to *O*-glycosylation profiling of human serum IgA, which possesses nine *O*-glycosylation sites at the hinge region [31,32]. Galactose depletion of *O*-glycans at the IgA hinge has been observed in the serum of patients with IgA nephropathy [31,32]. Figure 5a shows a typical elution profile on a Mono-Q column of the PA-*O*-glycans derived from the IgA sample, which were separated according to the degrees of sialylation. Each fraction was further separated on a C30 column as shown in Figure 5b. Individual fractions separated by the C30 column were further separated on an amide-silica column. The PA-oligosaccharides were identified on the basis of coincidence of the elution data with those in the HPLC map established in the present study. The incidence of *O*-glycan structures derived from serum IgA is indicated in Figure 5b. To date, IgA *O*-glycosylation has been characterized by lectin blotting, mass spectroscopy, and chromatographic separation [21,31,32,33]. These studies have described *O*-glycan structures Galβ1-3GalNAc, Neu5Acα2-3Galβ1-3GalNAc, Galβ1-3(Neu5Acα2-6)GalNAc-PA, and Neu5Acα2-3Galβ1-3(Neu5Acα2-6)GalNAc. To the best of our knowledge, the present study is the first to identify the di-sialyl *O*-glycan Neu5Acα2-6Galβ1-3(Neu5Acα2-6)GalNAc in serum IgA. The HPLC map developed in the present study enables us to distinguish the isomeric structures of sialyl *O*-glycans, offering quantitative information for *O*-glycosylation profiling.

## 4. Conclusions

In the present study, we developed an HPLC mapping method for detailed structural identification of *O*-glycans in neutral, sialylated, and sulfated forms. Furthermore, using this method, we were able to quantitatively identify isomeric products from an *in vitro* reaction catalyzed by human GlcNAc6ST-1 and obtain *O*-glycosylation profiles of human serum IgA as a model glycoprotein. The HPLC map will provide a glycomics tool for unambiguous identification and quantitative profiling of *O*-glycans expressed on a variety of proteins of physiological and pathological interest.
